# Potential Fungal Zoonotic Pathogens in Cetaceans: An Emerging Concern

**DOI:** 10.3390/microorganisms12030554

**Published:** 2024-03-11

**Authors:** Victor Garcia-Bustos, Begoña Acosta-Hernández, Marta Dafne Cabañero-Navalón, Alba Cecilia Ruiz-Gaitán, Javier Pemán, Inmaculada Rosario Medina

**Affiliations:** 1 University Institute of Animal Health and Food Security (ULPGC-IUSA), University of Las Palmas de Gran Canaria, 35416 Arucas, Spain; inmaculada.rosario@ulpgc.es; 2 Severe Infection Research Group, Health Research Institute La Fe, 46026 Valencia, Spain; marta.dafne.cabanyero@gmail.com (M.D.C.-N.); albacruiz@gmail.com (A.C.R.-G.); peman_jav@gva.es (J.P.)

**Keywords:** zoonotic diseases, fungal infections, marine mammals, cetaceans, public health, antifungal resistance, One Health, emerging fungal diseases

## Abstract

Over 60% of emerging infectious diseases in humans are zoonotic, often originating from wild animals. This long-standing ecological phenomenon has accelerated due to human-induced environmental changes. Recent data show a significant increase in fungal infections, with 6.5 million cases annually leading to 3.7 million deaths, indicating their growing impact on global health. Despite the vast diversity of fungal species, only a few are known to infect humans and marine mammals. Fungal zoonoses, especially those involving marine mammals like cetaceans, are of global public health concern. Increased human–cetacean interactions, in both professional and recreational settings, pose risks for zoonotic disease transmission. This review focuses on the epidemiology, clinical manifestations, and zoonotic potential of major fungal pathogens shared in humans and cetaceans, highlighting their interspecies transmission capability and the challenges posed by antifungal resistance and environmental changes. It underscores the need for enhanced awareness and preventative measures in high-risk settings to protect public health and marine ecosystems.

## 1. Introduction

Nowadays, more than 60% of new or re-emerging infectious diseases affecting humans, as well as all major pandemics in the last hundred years, are attributed to zoonotic origins, primarily wild animals [1]. Historically, the interchange of pathogens between species is an age-old ecological phenomenon, evident from ancient times with diseases like rabies, cholera, and the plague [2]. While many infections in animals are not inherently suited to infect humans, occasional cross-species transmission can lead to limited human outbreaks [1,3]. However, these diseases often do not adapt to new hosts, preventing widespread contagion [3]. Recent trends show an accelerating emergence of zoonotic diseases from wildlife, a development closely linked to human-induced environmental changes that increase interactions between humans, domestic animals, and wildlife [2,4].

Considering the escalating challenges posed by climate change, globalization, and ecological degradation, which are influencing the frequency and nature of zoonotic diseases, a radical transformation in our approach to prevention is crucial [1,5]. These climatic shifts are affecting interactions among animal hosts, disease carriers, and pathogens, thereby favoring the likelihood of new diseases emerging in human populations [6]. This scenario underscores a complex interplay between global human activities, the emergence of novel pathogens, and the degradation of ecosystems [4].

In this regard, fungal infections have historically been underemphasized in clinical practice and research [7]. Nonetheless, the emergence of new, alarming data underscores the urgent need for global attention and action in this field. A recent study published in *The Lancet Infectious Diseases* unveils updated figures on the prevalence of severe fungal infections [8]. The study reveals an alarming increase in these infections, with approximately 6.5 million cases annually, resulting in nearly 3.7 million fatalities. This contrasts sharply with previous estimates of 1.5 to 2 million cases per year. Considering the global annual death toll of around 55 million, fungal diseases could be responsible for or contribute to 6.7% of these deaths, exceeding the impacts of diseases like tuberculosis, malaria, hepatitis, and pneumonia. A critical aspect of this new estimate is discerning the direct fatalities caused by fungal infections versus deaths occurring in patients with other health complications. This analysis suggests that about 68% of these deaths are directly attributable to fungal diseases, though the range varies from 30% to 90% [8].

**Scheme 1:**
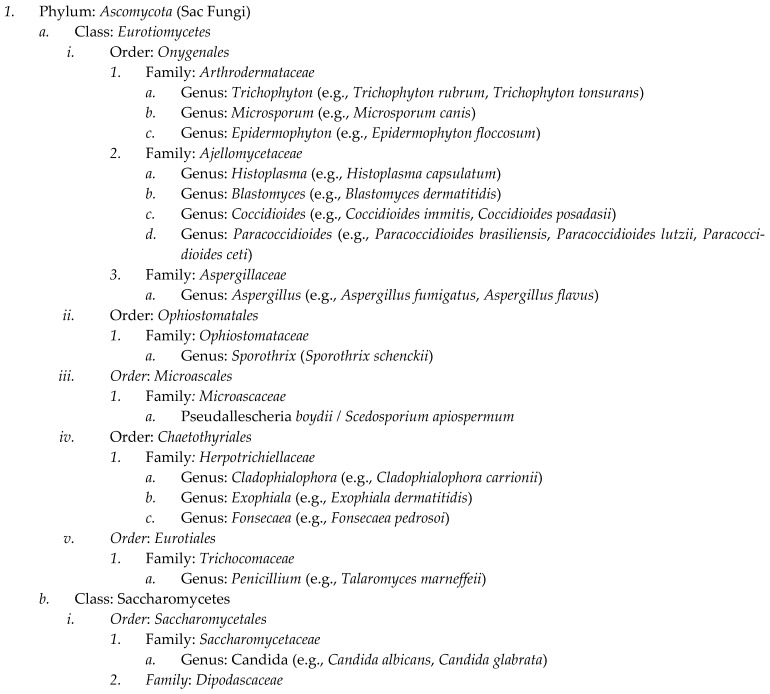

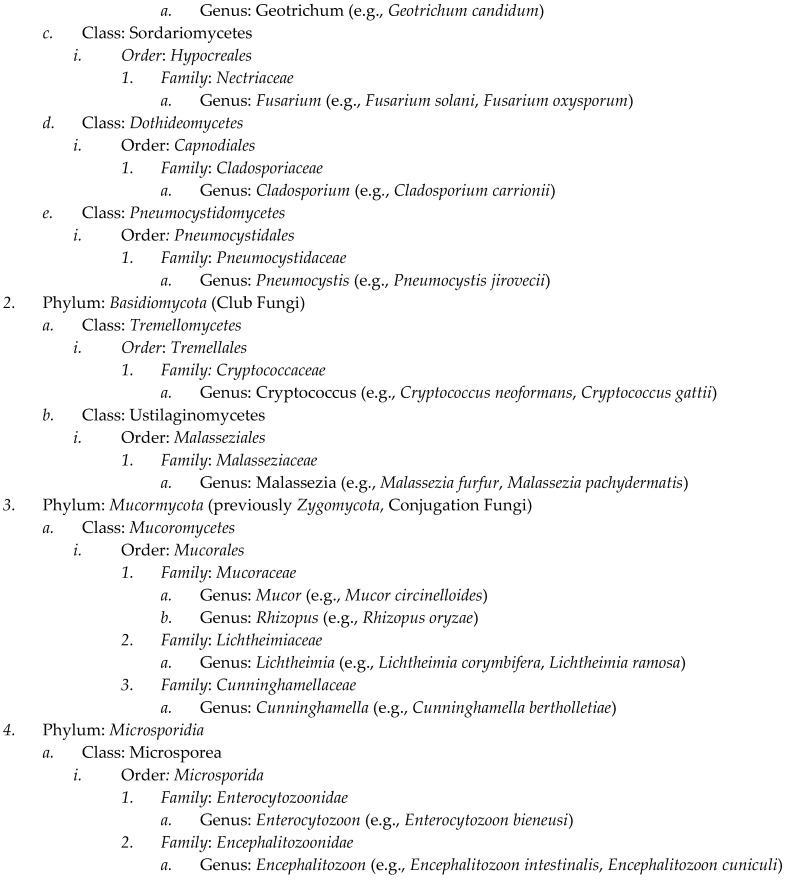
Comprehensive taxonomic classification of clinically relevant fungal species in mammals. To construct this phylogenetic tree encompassing clinically significant fungal species in mammals, we utilized the National Center for Biotechnology Information (NCBI) taxonomy database, alongside various other NCBI resources and databases, for comprehensive research and data compilation [24,25].

Currently, approximately 148,000 species of fungi have been identified. Advanced sequencing techniques suggest the existence of up to 5.1 million fungal species. Despite this vast diversity, only a select few species are commonly known to infect humans and other endothermic animals, including marine mammals [9]. The primary genera of pathogenic fungi are illustrated in the taxonomic tree shown in Scheme 1. A significant portion of these pathogenic species exhibit either zoonotic or sapronotic characteristics.

Fungal zoonoses, which involve the natural transmission of fungi between animals and humans, represent a significant global public health issue. Several mycoses known for their zoonotic transmission rank among the most prevalent fungal diseases worldwide [10]. Despite this, there is a noticeable disparity in international public health initiatives regarding certain fungal diseases with zoonotic capabilities. Additionally, this information gap could hinder the ability to foresee and manage emerging fungal pathogens. A pertinent example is *Candida auris*, which is theorized to have originated zoonotically from a marine environmental reservoir, with marine mammals potentially acting as intermediate hosts [11].

Cetaceans, as a group within marine mammals, hold a unique position in both the hearts of the public and in their ecological role as reservoirs of fungal zoonotic pathogens [12]. The general public’s fascination with these creatures has led to numerous interactive opportunities, such as whale-watching and dolphin-swimming programs, increasing human–cetacean contacts. The risk of zoonotic disease transmission in marine environments extends beyond public interactions with cetaceans, significantly affecting professionals, such as researchers, trainers, veterinarians, and volunteers. These individuals are particularly vulnerable due to their prolonged occupational exposure, a risk that is amplified during cetacean stranding events, where contact with infected carcasses is common [12]. However, an equally critical point of interaction, and perhaps more impactful, occurs in fisheries settings. In these environments, the intense workload and evolving fishing practices, including the incidental capture (bycatching) of cetaceans, lead to frequent and escalating contact in contexts that lack protective measures [13]. This increased interaction might present a substantial risk for the transmission of fungal zoonotic pathogens from cetaceans to humans. The implications of this transmission could be far-reaching, not only posing a direct threat to public health and straining healthcare systems, but also potentially impacting the safety and reputation of fish markets [14]. Such dynamics underscore the need for heightened awareness and preventative measures in these high-risk occupational settings to safeguard both public health and the integrity of the seafood industry.

The potential role of climate change and fungal diseases in cetaceans and, subsequently, humans, requires clarification. Associated with human activities, climate change can exacerbate the spread and severity of fungal diseases in marine mammals through several indirect pathways. Firstly, the potential emergence of new species acquiring thermal tolerance and pathogenicity, such as *C. auris*, might become a worrying problem in the near future [15]. Secondly, climate change is able to impact marine ecosystems in several ways, such as by increasing water temperature, changing oxygen contents and nutrient availability, altering oceanic pH levels, and causing water movements at a large scale, among others [16]. These changes affect the distribution and abundance of marine fungal pathogens, expanding the regions in which cetaceans are exposed to harmful pathogens [17,18,19]. These impacts are bidirectional, generating stress and immunocompromising wild cetacean populations, which make them vulnerable to fungal infections [20]. Furthermore, human activities such as pollution, also capable of deteriorating cetacean immune systems, and increased marine traffic can facilitate the spread of pathogens across different parts of the ocean, increasing the likelihood of cetaceans encountering disease-causing fungi [21,22]. This is also impacted by the change in cetacean migration patterns as a consequence of the deteriorating ecosystem effects of climate change and human activities. Their distribution to new areas in search of food or more suitable living conditions may imply increased opportunities for human–cetacean interactions, which could potentially lead to increased stress and susceptibility to diseases in these marine mammals [23].

Therefore, the aim of this review is to provide a comprehensive and updated overview of the epidemiology, clinical manifestations, and management strategies for major potential fungal pathogens affecting cetaceans. Special attention is given to the zoonotic capability of these pathogens; the challenges in diagnosis and treatment, particularly in the context of increasing antifungal resistance; and the impact of environmental changes, such as climate change and human activities, on the epidemiology of these fungal diseases. The review will be structured on the basis of each of the phyla contained in the previous scheme with zoonotic importance.

## 2. *Ascomycota*

In the following section, we delve into the crucial role of *Ascomycota* in humans and cetaceans. We will examine the main genera and species within this phylum, focusing on existing reports on cetaceans and discussing their potential as pathogenic agents and their zoonotic characteristics. 

### 2.1. The Blastomyces Genus

Blastomycosis represents a significant health concern in both terrestrial and marine settings, primarily caused by *Blastomyces* spp., a group of saprophytic dimorphic fungi belonging to the family *Ajellomycetaceae*. While historically endemic to specific regions, such as the Mississippi and Ohio River valleys and the Great Lakes region in North America, as well as parts of Africa, India, and the Middle East, the geographic distribution of *Blastomyces* has expanded, revealing a more complex epidemiological pattern [26,27,28].

In humans, the clinical presentation of blastomycosis ranges from asymptomatic to severe systemic involvement, with pulmonary manifestations being the most prevalent. The disease’s ability to mimic other conditions, such as community-acquired pneumonia and tuberculosis, complicates its diagnosis and underscores the necessity for robust epidemiological understanding and targeted diagnostic methods [26]. Treatment strategies vary based on disease severity, with itraconazole being standard for mild to moderate cases and amphotericin B reserved for more severe manifestations [26,29].

Remarkably, the zoonotic potential of *Blastomyces* from marine mammals has come to light through a documented case in infected Atlantic bottlenose dolphins (*Tursiops truncatus*), as evidenced by a treating veterinarian contracting cutaneous blastomycosis from an infected dolphin [30]. In this severe case, a bottlenose dolphin from the Gulf of Mexico developed extensive blastomycosis, beginning with an abscessed lesion and leading to widespread organ involvement [30]. Post-mortem analysis revealed significant yeast infiltration in the lungs, kidneys, and other vital organs, which was confirmed to be *B. dermatitidis* through specific immunofluorescent staining and immunodiffusion tests. Another report also identified blastomycosis in a dolphin, although specifics on the lesion were not provided [31]. These findings are not isolated; reports of blastomycosis in marine mammals such as Steller sea lions (*Eumetopias jubatus*) and California sea lions (*Zalophus californianus*) further exemplify the pathogen’s cross-species reach [32]. The clinical impact in marine mammals with systemic infections is manifested through a range of unspecific clinical symptoms, including depression, weakness, and anorexia, potentially culminating in death. Pathological examination often reveals pyogranulomatous inflammation across multiple organ systems, with the presence of the characteristic large round yeast cells of *Blastomyces* [33,34].

The discovery of cryptic species within the *Blastomyces* genus, such as *B. gilchristii* and *B. helicus*, and the recognition of distinct genetic populations within *B. dermatitidis*, underscore the complex nature of this fungal group and its potential for varied virulence, ecological niches, and geographic distribution [27,28,35]. The identification of *B. percursus* from patients in South Africa and Israel further expands the known range of *Blastomyces* species, suggesting a broader global presence and more varied pathogenic potential than previously understood [36].

This fact signals a potential expansion of the threat posed by *Blastomyces* to both cetaceans and humans. This genetic diversity indicates not only a broader geographic distribution but also varying disease severities and ecological impacts. For cetaceans, this means a heightened risk of exposure and potential challenges in accurately diagnosing and effectively treating infections, necessitating more vigilant surveillance and advanced understanding of the pathogen’s behavior in marine environments [27,28,35,36].

### 2.2. The Histoplasma Genus

*Histoplasma capsulatum*, traditionally associated with soil enriched by avian or bat droppings, poses a multifaceted threat due to its capacity to infect a wide array of hosts, including humans and cetaceans. This fungus, notorious for causing histoplasmosis, often manifests in severe forms among immunocompromised individuals [37,38]. While it is predominantly found in terrestrial environments, notably within the Mississippi and Ohio River Valleys and certain areas of Central and South America, in recent decades, there has been a notable rise in the incidence of infections caused by endemic fungi in regions traditionally considered outside of their endemic zones [39]. Furthermore, evidence suggests that its prevalence extends into marine ecosystems and that marine mammals could be also affected, particularly Atlantic bottlenose dolphins along coastal regions, as suggested by previous reports [40,41].

Climate change and human alteration of land have reshaped the habitats favorable to *H. capsulatum*, consequently shifting the disease’s epidemiological patterns [39]. In humans, histoplasmosis presents a challenging diagnostic landscape due to its wide array of clinical symptoms, which can mimic conditions such as community-acquired pneumonia, tuberculosis, sarcoidosis, Crohn’s disease, and even malignancies. Accurate diagnosis hinges on a comprehensive grasp of the disease’s epidemiology, typical clinical manifestations, and the most effective diagnostic approaches. While many cases in immunocompetent individuals are subclinical or resolve spontaneously and may not necessitate treatment, it is crucial to administer therapy to immunocompromised patients, those with progressive disseminated histoplasmosis, or chronic pulmonary forms of the disease. For severe or disseminated histoplasmosis, liposomal amphotericin B is the treatment of choice, whereas itraconazole serves as an effective option for less severe cases and as a continued treatment after initial response to amphotericin B [37,38]. 

Recent findings reveal that *H. capsulatum* can induce disseminated histoplasmosis in cetaceans, an infection pattern notably similar to human cases. For example, two bottlenose dolphins were diagnosed with histoplasmosis through both molecular methods (PCR) and serum antigen testing. Intriguingly, retrospective serum assays indicated prolonged exposure to *H. capsulatum*, hinting at a latent infection that could precede active disease states [40], as occurs in humans. A comprehensive 30-year retrospective study highlighted the prevalence of pneumonia in captive bottlenose dolphins, with one case of severe disseminated infection linked to *H. capsulatum*, emphasizing the pathogen’s potential impact on marine mammal health even in captive populations [41].

The occurrence of *H. capsulatum* in marine mammals not only raises concerns about the pathogen’s environmental resilience and adaptability but also underscores potential zoonotic pathways. Cetaceans frequenting coastal areas may encounter and harbor the pathogen. The implications for cetacean health could be significant, especially considering the changing epidemiology of these infections [42]. Further research in stranded networks should also focus on diagnosing these fungal infections mainly in endemic regions, as many of them might be misdiagnosed and might reveal an unknown problematic.

### 2.3. The Coccidioides Genus

Coccidioidomycosis, commonly known as Valley Fever, is an emerging concern in both terrestrial and marine ecosystems [43,44]. This invasive mycosis, primarily caused by *Coccidioides immitis* and *C. posadasii*, is traditionally known to be endemic to arid soils of the southwestern US, parts of Mexico, and Central and South America. However, the fungus demonstrates remarkable resilience in saline environments, such as seawater, expanding its potential impact beyond traditional geographical boundaries [43,44,45]. Furthermore, as with the previously discussed dimorphic fungi, these species are also experiencing an expansion of their geographical distribution [46]. 

The infection route for *Coccidioides* involves the inhalation of arthroconidia, which become airborne following both natural phenomena and anthropogenic activities. Recent observations indicate an increasing incidence and expanding geographic distribution of *Coccidioides*, with cases now reported in previously unrecognized areas, such as eastern Washington, Oregon, and Utah. While most infections are asymptomatic, a significant proportion develop into pulmonary illness, contributing notably to community-acquired pneumonia in endemic regions. Furthermore, dissemination to extrapulmonary locations occurs, especially among individuals with compromised cellular immunity [43,44].

The zoonotic and environmental impact of *Coccidioides* extends to marine wildlife, as highlighted by a case in 1995 involving a wild adult female bottlenose dolphin in California. The dolphin, infected with *C. immitis*, exhibited severe dyspnea, and underwent rapid clinical deterioration, leading to death from pneumonia. Post-mortem examination confirmed the presence of *C. immitis* in the lungs, lymph nodes, and brain, underlining the pathogen’s ability to thrive in marine hosts and environments [47]. This case, coupled with documented infections in California sea lions and sea otters (*Enhydra lutris*), marks a significant extension of *Coccidioides* into marine species [48].

Adding to the concern, a study by Kanegae et al. (2022) [49] reported a 15.4% seroprevalence against *C. posadasii* in porpoises (*Phocoenoides dalli* and *Phocoena phocoena*) stranded in Hokkaido, Japan, indicating a broader environmental and host range than previously understood. These findings underscore the adaptability of *Coccidioides* and the potential risks it poses not only to humans but also to marine wildlife. 

### 2.4. The Paracoccidioides Genus

Paracoccidioidomycosis ceti, previously termed lacaziosis or lobomycosis, represents a significant health concern in cetaceans, and its reclassification has pivotal implications for our understanding of zoonotic diseases from cetaceans. Initially described by Jorge de Oliveira Lobo in 1931 in humans, and later in dolphins in 1971, this disease has been subject to extensive taxonomic review and molecular scrutiny. The uncultivable pathogens in dolphins and humans causing this condition have been identified as distinct species: *Paracoccidioides ceti* in cetaceans and *Paracoccidioides lobogeorgii* in humans [50,51,52,53,54]. This differentiation is crucial, as it challenges the prior assumption of a direct zoonotic relationship between the diseases in dolphins and humans. While reports of human transmission from dolphins have previously been described, such instances are infrequent [55,56,57], emphasizing the need for careful interpretation of zoonotic implications, particularly given the recent taxonomic distinctions. Cases of paracoccidioidomycosis ceti have been reported globally, with most dolphin infections noted along Florida’s coastline [58] and additional cases in the eastern and western Atlantic, the Pacific, and Indian Oceans. Significantly, reports of the disease extend beyond the Americas to countries like France, Spain, Madagascar, South Africa, and Japan, although all human cases in non-endemic countries were imported [55,59,60,61,62,63,64,65,66].

Paracoccidioidomycosis ceti manifests through distinct clinical features influenced by environmental factors. Typical lesions are raised, adopting a nodular or verrucous profile, and may ulcerate or expand into plaques. Anatomical areas commonly affected include the dorsal cranial surface, anterior dorsum, and fins. Pathologically, the disease is characterized by acanthosis, hyperkeratosis, hyperpigmentation, fibrosis, lymphohistiocytic infiltration, and microabscesses filled with yeast-like cells [58,66,67,68,69,70].

The disease’s diagnosis is complicated by many reports relying solely on phenotypical lesion characterizations, without proper microbiological identification of the etiological agents. This challenge is compounded by the taxonomical complexity historically surrounding the disease’s definition and the potential for other fungal pathogens to cause similar cutaneous conditions [59,60,64,68]. 

Given these complexities, adherence to a precise taxonomy and nomenclature is essential. The shift from “lobomycosis” to paracoccidioidomycosis lobogeorgi for *P. lobogeorgii* infections in humans and paracoccidioidomycosis ceti for *P. ceti* infection in cetaceans reflects a commitment to scientific accuracy and clarity in communication, as reported in the recent letter by Vilela and Mendoza [67]. It honors the research legacy and is critical for effective disease management and the interpretation of zoonotic implications [71].

This disease is an important consideration in cetacean conservation due to its prevalence in dolphin populations; the severity of the chronic skin lesions, which can be debilitating for infected dolphins, potentially affecting their health and survival; and the current limitations in treatment and understanding of the disease’s transmission. The impact on dolphin health and the potential for spread within marine environments underscore the need for ongoing research and conservation efforts.

### 2.5. The Candida Genus

*Candida* species are known for their dual role as both commensals and pathogens. They are common inhabitants of mucosal surfaces in humans and various animal species, including marine mammals. Under certain conditions, such as immunosuppression or disruption of the normal microbiota, *Candida* can transition from a harmless commensal to a pathogenic state, leading to infections ranging from superficial mucocutaneous diseases to life-threatening systemic conditions [72,73].

In human medicine, *Candida* infections have garnered increasing attention due to their rising incidence, particularly in immunocompromised individuals and hospitalized patients. These infections are concerning due to the significant morbidity and mortality they can cause, as well as the growing problem of antifungal resistance, which complicates treatment options and leads to poorer patient outcomes [10,74]. Around 1.5 million people die of invasive fungal infections every year worldwide. About 80% of deaths caused by fungal sepsis are due to hospital-acquired opportunistic fungal infections, most caused by *Candida* species [75]. Invasive candidiasis encompasses candidemia and infections in normally sterile sites. Various conditions and treatments, such as malignancies, surgeries, transplantations, immunosuppression, and prolonged hospital stays, particularly in intensive care units, are risk factors for these infections [76].

Among the numerous *Candida* species, *C. albicans*, *C. glabrata*, *C. krusei*, *C. tropicalis*, and *C. parapsilosis* are the most common, accounting for over 90% of invasive infections [77]. Recently, *C. auris* has emerged globally as a notable pathogen due to its multi-drug resistance, its ability to form biofilms and persist on surfaces, and its high nosocomial transmission potential [78], which has been associated with climate change [11].

The therapeutic arsenal against candidiasis includes barely three classes of antifungal drugs: polyenes, azoles, and echinocandins, with each of them having issues that limit their clinical use [79]. For example, polyenes can cause severe nephrotoxicity due to the non-selective targeting of mammalian membrane cholesterol [80]. A limited antifungal spectrum, a requirement of intravenous administration, and high drug costs have become challenges for the clinical use of echinocandins [80]. Azoles have only fungistatic effects in *Candida* species, which leads to the emergence of azole-resistant isolates [74,80].

The occurrence of *Candida* infections in cetaceans offers an interesting parallel to human candidiasis. Although historically understudied compared to bacterial and viral diseases in marine mammals, fungal infections, particularly those caused by *Candida* spp., have been increasingly reported in both captive and wild cetacean populations [21,48,81]. These infections manifest various clinical signs, including respiratory tract infections, skin lesions, and even systemic involvement, which can have profound implications for the health and conservation of these marine species [31,33,82].

*C. albicans*, a commonly reported species, has been implicated in a wide array of conditions in these animals. Chronic cutaneous candidiasis in captive performing bottlenose dolphins [82] and visceral systemic candidiasis in deceased killer whales (*Orcinus orca*), affecting multiple organs, including the heart, kidneys, and lymph nodes, highlight the potential severity of infections [33]. Additionally, *C. albicans* has been identified as the causative agent in various cetaceans, including an Atlantic bottlenose dolphin, a beluga whale (*Delphinapterus leucas*), a juvenile harbor porpoise (*P. phocoena*), and a pilot whale (*Globicephala melas*) [83]. Systemic candidiasis with non-specific symptoms such as lethargy and erratic behavior, coupled with respiratory and digestive symptoms, was reported in captive dusky dolphins (*Lagenorrhynchus obscurus*) in South Africa. The presence of *C. albicans* in the blowholes, mouths, tongues, feces, or gastric contents of these dolphins signifies its pervasive nature [84].

*Candida tropicalis* is another significant species identified in cetaceans. Its presence in captive dolphins, including false killer whales (*Pseudorca crassidens*), bottlenose dolphins, and F1 offspring between bottlenose dolphins and Pacific bottlenose dolphins, underscores the widespread occurrence of *Candida* spp. in marine environments [85]. The similarity of genotypes of *C. tropicalis* in dolphins, environments, and even among staff members in an aquarium setting highlights the potential for interspecies transmission and environmental contamination.

*Candida glabrata*, known for its intrinsic resistance to azoles, represents a critical challenge due to its antifungal resistance. It has been isolated from various cetacean species, including a US navy bottlenose dolphin, where it caused a chronic cervical abscess necessitating surgical intervention [86]. Additionally, respiratory co-infection with *C. glabrata* and parainfluenza virus in a bottlenose dolphin in the USA [87], and its presence in wild cetaceans demonstrates the adaptability and virulence of this species.

The occurrence of other *Candida* species, such as *C. krusei*, *C. parapsilosis*, *C. guilliermondii*, and *C. lambica*, in various anatomical sites of cetaceans [84,88,89] broadens the spectrum of fungal pathogens affecting these marine mammals. The emergence of antifungal resistance, as evidenced by the resistance patterns of *Candida* spp. isolated from cetaceans, poses a significant public health concern. The association of higher prevalence of azole antifungal resistance with increased halotolerance [90] and the link between the pathogenicity of various *Candida* species and increasingly severe environments and climate change [23] call for vigilant monitoring and effective management strategies.

In wild cetacean populations, the presence of *Candida* spp. as part of the normal tissue microbiota, as observed in bowhead whales (*Balaena mysticetus*) [91], along with the identification of various *Candida* species in stranded Atlantic white-sided dolphins (*Lagenorhynchus acutus*) [92], underscores the ecological significance of these fungi in the wild. 

According to these data, evidence suggests that controlling *Candida* infections is important for cetacean conservation due to their recognized impact on cetacean health, from superficial to life-threatening systemic infections, severe respiratory infections, and skin lesions, impacting both healthy and immunocompromised individuals.

The occurrence of *Candida* spp. in free-ranging bottlenose dolphins, with varying prevalence across different species, highlights the need for further research to understand the impact of these fungi on wild cetacean health and their potential role in ecosystem dynamics [93].

The zoonotic potential of *Candida* species from cetaceans, particularly in the context of human–cetacean interactions, warrants careful consideration. The documentation by Buck (1980) of human-associated yeasts in the feces and pool waters of captive bottlenose dolphins underscores a significant overlap between human and cetacean microbiota [94]. This overlap suggests a possible route for zoonotic transmission, given the shared environment and close contact in captivity. Furthermore, the adaptability and persistence of *Candida* species in aquatic environments may facilitate their transmission between species.

### 2.6. The Fusarium Genus

*Fusarium* spp. are increasingly recognized for their significant implications in human medicine, primarily due to their role as opportunistic pathogens capable of causing a spectrum of infections, particularly in immunocompromised individuals [95,96]. In agriculture, *Fusarium*’s phytopathogenic properties are notorious for contributing to global food scarcity and undernutrition [95]. However, in a clinical context, species such as *F. oxysporum* and *F. solani* are emerging as notable threats to human health, causing localized infections of nails, skin, and sinuses, and occasionally leading to severe, systemic infections, with a poor response to available systemic antifungals [95,96].

In cetaceans, *Fusarium* spp. have been identified as opportunistic fungal pathogens, exhibiting a range of clinical manifestations. Instances of raised, firm, erythematous cutaneous nodules were observed in an Atlantic white-sided dolphin (*Lagenorhynchus acutus*), a pygmy sperm whale (*Kogia breviceps*), and two harbor seals (*Phoca vitulina*), with microbial culturing isolating *Fusarium* spp. [97]. These nodules resolved 3 to 4 weeks post-treatment with ketoconazole. A case involving an adult male beluga whale presented a nodule that evolved into a cutaneous ulcer, with PCR confirming *F. solani* as the causative agent [98]. Despite initial drug resistance, a regimen including surgical debridement, topical and regional antifungals, and oral voriconazole resulted in lesion improvement, emphasizing the complexity and adaptability of *Fusarium* infections. Furthermore, in a female false killer whale (*Pseudorca crassidens*), *F. solani*-induced lesions penetrated the deep dermis and bone, an uncommon presentation for *Fusarium* spp. infections in marine mammals [99]. The lesions comprised multiple granulomas with fungal hyphae and chlamydospores, with a more invasive behavior. In this connection, a necropsy on a male Atlantic bottlenose dolphin revealed acute necrotizing meningoencephalitis with intralesional fungal hyphae, with cultures and sequencing confirming *F. oxysporum* as the causative agent [100]. This case represents a rare instance of non-cutaneous *Fusarium* infection in a cetacean, indicating the potential for the pathogen to cause severe systemic infections similar to disseminated infections in immunocompromised humans.

Environmental studies at the Port of Nagoya Public Aquarium revealed the presence of various fungi, including *Fusarium* spp., in the pool and air, though these were not directly linked to the fungal infections in two deceased killer whales at the facility [101]. This highlights the potential environmental reservoirs of *Fusarium* spp. and other fungi in aquatic settings. The documented infections in cetaceans, alongside the presence of these pathogens in shared aquatic environments and their known pathogenicity in humans, particularly in immunocompromised individuals, highlight the potential for zoonotic transmission and underscore the importance of vigilant environmental monitoring and robust clinical awareness of *Fusarium*-related diseases.

### 2.7. The Aspergillus Genus

*Aspergillus* is a genus of filamentous fungi comprising numerous species of clinical and environmental importance. In humans, it is notably implicated in a range of conditions known as aspergilloses, with manifestations varying from non-invasive allergic reactions to severe invasive pulmonary infections, influenced by the host’s immune responses [102,103,104]. The ubiquitous nature of *Aspergillus* conidia, coupled with their potential to initiate localized or systemic infections post-inhalation or -inoculation, poses significant health risks, particularly in the context of host immune states [102,103,104]). The emergence of azole resistance in *Aspergillus* spp. worldwide, attributed to environmental pressures and the escalation of long-term azole prophylaxis and treatment, especially in immunocompromised patients such as hematopoietic stem cell transplant recipients, has exacerbated these risks [103,105]. The management of aspergillosis presents multifaceted challenges, including the rise of multidrug-resistant strains, adverse drug interactions, side effects, and patient-specific conditions [102]. 

In the animal kingdom, aspergillosis predominantly manifests as a respiratory infection with the potential to disseminate, although its affinity for specific tissues varies across different species. Mirroring the human scenario, animals with compromised immune responses are more susceptible to aspergillosis. Furthermore, even animals in good health can succumb to the infection when subjected to environmental stressors or conditions that weaken their immune defenses [10]. Furthermore, *Aspergillus* can be transmitted from animals to humans, as can other zoonotic fungi, mostly following environmental exposure [10,106].

*Aspergillus* infections in cetaceans present a noteworthy aspect of marine mammal pathology, with multiple cetacean species being affected by different species of the genus. *Aspergillus fumigatus*, in particular, has been frequently identified as a major causative agent in a variety of cetacean species, similar to humans.

In the Atlantic spotted dolphin (*Stenella frontalis*), cases of pulmonary aspergillosis have been reported, one involving localized pulmonary disease with pyogranulomatous and necrotizing bronchopneumonia [107,108] and another displaying chronic inflammation of the meninges, brain, and spinal cord along with suppurative fungal tracheitis and bronchopneumonia [108]. Similarly, the bottlenose dolphin has been a subject of concern with instances of disseminated coinfection of dolphin morbillivirus and *A. fumigatus* [109], as well as cases involving pneumonia and cerebral lesions due to this species [110,111,112,113,114]. Additionally, azole-resistant *A. fumigatus* has been identified as a challenge in the treatment of invasive aspergillosis in bottlenose dolphins [114].

Other cetacean species, such as the Bryde’s whale (*Balaenoptera edeni*), have been reported to suffer from systemic mycosis involving *Aspergillus* spp., particularly with orchitis, periorchitis, mesenteric lymphadenitis, and pyogranulomatous bronchopneumonia [107]. Killer whales have been reported to suffer disseminated aspergillosis, with environmental studies suggesting the presence of *Aspergillus* spp. in their aquatic habitats [101,115].

In harbor porpoises, *Aspergillus* infections have manifested as severe mycotic otitis media [116,117] and intracranial granuloma [118]. The northern bottlenose whale (*Hyperoodon ampullatus*) also exhibited a case of fungal encephalitis due to *A. fumigatus* [119,120]. The striped dolphin (*Stenella coeruleoalba*) is another species in which *A. fumigatus* has been implicated in occlusive mycotic tracheobronchitis and other respiratory conditions [121,122]. A rare case was reported in which *A. lentulus* was isolated from a dolphin nostril, though the specific species of the cetacean was not detailed [123,124]. The diversity in *Aspergillus* spp. infections across different cetacean species underscores the need for vigilant monitoring and innovative treatment approaches, particularly in the context of rising antifungal resistance and the complexity of these infections in marine mammals.

The detection of *Aspergillus*-related infections in cetacean populations, coupled with the established prevalence of this fungus in human infections, suggests a zoonotic potential that necessitates closer examination. *Aspergillus fumigatus* has been identified as a common pathogen in various cetacean species, reflecting patterns of disease similar to those seen in humans. The occurrence of this fungus in marine habitats and the emergence of azole-resistant strains across both cetacean and human cases underscore the importance of an integrated health approach. Furthermore, aspergillosis significantly impacts cetacean conservation by causing respiratory infections that lead to increased morbidity and mortality, thus necessitating targeted health management strategies within these marine mammal populations beyond the captive environment.

## 3. *Basidiomycota*

### 3.1. The Cryptococcus Genus

Cryptococcosis has become a critical systemic fungal disease, particularly pronounced in areas heavily impacted by the HIV/AIDS crisis, such as Sub-Saharan Africa. In these regions, the disease is further deepened due to limited healthcare accessibility and widespread cases of advanced immunodeficiency [125]. This infection, predominantly instigated by the fungi *Cryptococcus neoformans* and *Cryptococcus gattii*, now stands as the third most prevalent condition among HIV-positive individuals. Estimates suggest that there are approximately one million cases of cryptococcal meningitis annually among patients with AIDS [125,126,127]. In the human clinical setting, *C. neoformans* primarily affects immunocompromised individuals, leading to complications in the central nervous system. Conversely, *C. gattii* is known to cause severe pulmonary diseases, which can occur in both immunocompromised and immunocompetent individuals. A notable challenge in medical diagnostics is pulmonary cryptococcosis, a type of invasive lung mycosis that is often misdiagnosed as pulmonary malignancy, leading to delays in appropriate treatment [127]. This underscores the importance of reliable diagnostic techniques, including histopathology, microscopy, and culture, along with the detection of cryptococcal polysaccharide antigens or *Cryptococcus*-derived nucleic acids [125].

The management of cryptococcosis requires an extensive treatment regimen, beginning with an induction phase using amphotericin B and flucytosine, followed by consolidation therapy with fluconazole. The treatment process is complicated by the growing presence of azole-resistant *Cryptococcus* strains, making susceptibility testing vital, particularly in areas reporting high minimum inhibitory concentrations to fluconazole [125,126,127].

Cryptococcosis’s epidemiology is influenced by environmental factors and human activities. This environmental pathogen now thrives in diverse habitats, including soil, trees, birds, and domestic pets [128]. The disease has been noted in association with reticuloendothelial cancers since the 1950s and has gained prominence with the advent of transplant immunology, solid organ and hematological stem cell transplantations, and the use of biological immunotherapeutics. Environmental outbreaks of both human and animal cryptococcosis, potentially driven by climate change, have been reported, exemplified by the resurgence of *C. gattii* infection in Vancouver Island, Canada, and the Pacific Northwest of the United States since 1999 [128].

Cryptococcosis affects not only humans but also a broad spectrum of animal hosts, from *Acanthamoeba* to large mammals, presenting as both overt diseases and subclinical infections in diverse wildlife, pets, livestock, and marine mammals [129,130,131]. This zoonotic aspect of cryptococcosis highlights the importance of continuous surveillance and the need for innovative treatment approaches in the face of rising antifungal resistance and complex infection dynamics in both human and animal populations.

In this regard, it poses a significant health threat to cetacean species. *Cryptococcus* spp., unlike other fungal species, are not typical colonizers in cetaceans and are primarily linked to invasive diseases with high mortality rates [132]. Cetaceans’ susceptibility to *Cryptococcus* is particularly notable during coastal migrations, in captivity, or when in proximity to terrestrial regions where infectious propagules from effluents and runoff enter marine environments [129]. These infections can occur as outbreaks and are often associated with detections in humans and other animal species [133]. Notably, the emergence of *C. gattii* in North America in 1999 led to a multispecies cryptococcosis outbreak in British Columbia, Washington State, and Oregon, significantly impacting marine mammals [132,133,134]. The transmission primarily occurs through the inhalation of basidiospores. The absence of sinonasal filtration mechanisms in cetaceans, coupled with their large tidal volume, allows these infective propagules to reach the lower respiratory system [129]. Pneumonia, often escalating to disseminated systemic infections, is the predominant clinical manifestation in cetaceans [48,135]. Generalized lymphadenopathies and multiorgan affectation can also occur [70,136]. A unique case of maternal–fetal transmission of *C. gattii* in a harbor porpoise has been reported [137].

Cryptococcal invasive disease affects both wild and captive cetaceans. *C. neoformans* infections have been identified in baleen whales, such as the southern right whale (*Eubalaena australis*), and odontocetes, including Dall’s and harbor porpoises (*P. dalli* and *P. phocoena*, respectively), primarily in necropsies of stranded animals [41,133,138,139]. *Cryptococcus gattii* infections have impacted various species, such as bottlenose dolphins, spinner dolphins (*Stenella longirostris*), Dall’s and harbor porpoises, and Pacific white-sided dolphins (*Lagenorhynchus obliquidens*) [41,70,132,133,134,135]. These cases have been reported worldwide, in the Atlantic, Pacific, and Indian Oceans, from British Columbia to South Africa and Western Australia. However, the understanding of *Cryptococcus* as a non-colonizing organism in cetaceans is being challenged by recent findings. Investigations employing culture-based or molecular methodologies revealed the presence of *Cryptococcus* spp. in skin samples from bowhead whales (*Balaena mysticetus*) and gastrointestinal microbiota of East Asian finless porpoises (*Neophocaena asiaeorientalis sunameri*) [91,140]. 

The literature on cryptococcosis in cetaceans varies from single case studies to more comprehensive examinations, with variable reliability depending on the diagnostic specimens and tools used. Notably, antifungal susceptibility data are scarce, with only one reported case detailing minimum inhibitory concentrations to itraconazole in a bottlenose dolphin [135]. Anthropogenic activities, such as construction and deforestation, are thought to contribute to the incidence of cryptococcal disease in cetaceans and other species by disturbing soil and prompting aerosolization of *Cryptococcus* spores [132]. The severity of these infections and the possibility of outbreaks, such as those seen in North America in the context of climatic events, highlight the urgent need for ongoing research and monitoring to protect cetaceans. This situation emphasizes the critical role of cetaceans in indicating the health of marine ecosystems and the importance of their conservation in the face of changing global conditions.

### 3.2. The Malassezia Genus

*Malassezia* species, commonly found as commensals on the skin, in the oral and sinonasal cavities, as well as in the lower respiratory and gastrointestinal tracts, encompass eighteen species that inhabit humans, various mammals, and birds [141,142,143]. These species have been recovered from multiple environments, indicating their evolutionary adaptation from an original ecological niche in plants and soil to the mucocutaneous ecosystem of warm-blooded vertebrates [141]. In both humans and pets like dogs and cats, dermatological conditions associated with *Malassezia* exhibit certain similarities. While otomycosis is frequently observed in companion animals, it is comparatively rare in humans. Systemic infections by *Malassezia*, increasingly reported in human cases, have not yet been recognized in the animal population. Furthermore, *Malassezia* species have been identified as contributing factors in certain chronic human diseases [141,142,143].

Some species in the genus, while primarily host-adapted, display zoophilic characteristics and have been implicated in cases of fungemia. Notably, outbreaks in neonatal intensive care units have been linked to healthcare workers’ transient hand colonization after contact with their pets [141,144]. The susceptibility to antifungals can differ based on several factors, including the specific *Malassezia* species, the location of infection on the body, the type and duration of the infection, the presence of co-morbidities, and the state of the host’s immune system [142,143].

Despite the zoonotic importance of these infections, there is a complete lack of evidence in cetaceans. While this pathogen has been associated with cutaneous disease in marine mammals such as pinnipeds [145,146], there is no culture-based evidence of *Malassezia*-associated disease in purely aquatic animals such as cetaceans. However, in a study using high-throughput sequencing for fungal community at the genus level in the gastrointestinal tract of East Asian finless porpoises, *Malassezia* spp. were highly abundant in the foregut [140]. Furthermore, a study investigating the oral and genital fungal microbiome of eight dolphins stranded on the coast of Portugal, utilizing molecular techniques, revealed that *Malassezia* species were predominant in both niches. Given these recent findings, additional research is essential to enhance our understanding of these species in these hosts.

## 4. *Mucormycota*

Mucormycosis is an emerging and often fatal fungal infection caused by fungi from the *Mucorales* family. The most commonly reported causative agents are *Rhizopus* spp., *Mucor* spp. and *Lichtheimia* spp. (formerly known as *Absidia* and *Mycocladus*), followed by *Rhizomucor* spp., *Cunninghamella* spp., *Apophysomyces* spp. and *Saksenaea*. spp. The first three species account for over 90% of all mucormycosis cases [147,148,149]. In humans, this infection affects patients with significant immunosuppression, including those diagnosed with hematologic malignancies or who have undergone organ transplants. Additionally, individuals with diabetes mellitus and, characteristically, in situations of diabetic ketoacidosis are particularly vulnerable. The recent surge in COVID-19 cases has further escalated the incidence of mucormycosis, especially among those with poorly controlled diabetes and patients undergoing corticosteroid therapy [147,148,149]. Clinically, mucormycosis manifests in various forms, such as rhino-orbital/rhino-cerebral mucormycosis, sino-pulmonary infections, and necrotizing skin conditions [147,148,149]. Effective management of mucormycosis necessitates prompt recognition and initiation of antifungal therapy specifically active against *Mucorales*. Strategies also include reversing the underlying causes of immunosuppression, swiftly correcting diabetic ketoacidosis, and considering surgical removal of infected tissue in select cases. The first-choice therapy usually involves high-dose liposomal amphotericin B. Additionally, intravenous isavuconazole and posaconazole, administered either intravenously or as delayed-release tablets, are recommended, particularly as second-line treatments [148].

In both humans and animals, including domestic, wild, mammalian, and non-mammalian species, the patterns of mucormycosis infection are remarkably similar in terms of epidemiology, mode of entry, site of infection, and lesion development [10]. Pathogenic *Mucorales* species, known for their opportunistic nature, are commonly found in environments frequented by domestic animals and within indoor settings. Infections generally arise when there is a disruption in the typical equilibrium between the animal and the fungal agent [10]. Predisposing factors for mucormycosis in animals are akin to those in humans, hinging not on the species but on the specific health status of the individual animal. This includes states of compromised immunity or metabolic imbalances. Nonetheless, intense exposure to these fungi or changes in the normal microbiome, particularly in the forestomach of certain animals, can precipitate infection even in otherwise healthy individuals [10]. 

Mucormycosis in cetaceans is an emerging concern in marine mammal health. This fungal infection has been documented in several cetacean species, exhibiting a range of clinical manifestations and high mortality rates. In a study spanning a decade, mucormycosis caused by *Saksenaea vasiformis* and *Apophysomyces elegans* was reported in a killer whale, two Pacific white-sided dolphins, and two bottlenose dolphins housed at SeaWorld in Texas. These cases typically involved the subcutaneous tissue and skeletal musculature, with systemic spread noted in some instances, including to the central nervous system [150]. 

A case of *Cunninghamella bertholletiae*, a thermophilic species causing human mycoses, was identified in a central nervous system infection in a bottlenose dolphin [151]. Another report involved pulmonary mucormycosis with *C. bertholletiae* in a killer whale [115]. Moreover, a bottlenose dolphin with central nervous system mucormycosis due to *C. bertholletiae* was recorded, further emphasizing the potential severity of such infections in cetaceans [152]. These reports add to the scarce information about *C. bertholletiae* infections in animals. 

Systemic mucormycosis caused by *Rhizopus microsporus* in a captive bottlenose dolphin, characterized by disseminated fungal pyogranulomas in various organs, was also documente [153]. An instance of pyogranulomatous obliterative laryngotracheitis by *Rhizopus arrhizus* (syn. *R. oryzae*) in a free-ranging Atlantic spotted dolphin highlighted another species of *Mucorales* affecting cetaceans [154]. A harbor porpoise from the Baltic Sea was also affected by *Rhizopus* spp., presenting with systemic mycosis and granulomatous mycotic lesions in multiple organs [155]. 

Additionally, disseminated mycosis in a killer whale involving dual infection with *Mucor* and *Aspergillus* species was reported, providing insight into the complexity of fungal infections in these animals [115]). Finally, the successful treatment of suspected mucormycosis in a bottlenose dolphin calf using posaconazole was reported, providing a potential therapeutic avenue for managing this challenging condition [156].

These reports collectively underscore the need for increased awareness and research into mucormycosis in cetaceans, considering the varied etiological agents and the severe impact on the health of these marine animals. The presence of mucormycosis in these animals indicates a potential environmental reservoir for these fungi, which can infect humans, especially those with weakened immune systems. While direct zoonotic transmission from cetaceans to humans is unlikely due to limited contact and the need for combined immune suppression in different hosts, these findings underline the importance of monitoring fungal diseases in marine wildlife and their potential implications for marine mammal conservation.

## 5. Other Reports

Several fungi with a widespread environmental presence and the capacity for dimorphic behavior in some of them have been occasionally documented as causing skin and soft tissue infections, often involving the lymphocutaneous region. In some instances, these fungi can lead to deep-seated infections, particularly in immunocompromised individuals among both animals and humans. While infrequently reported, there have been marginal observations of such infections in cetaceans. These fungi include *Cladosporium*, *Conidiobolus*, and *Sporothrix.*

In cetaceans, *Cladosporium* species have been identified in the feces of free-ranging sperm whales in the Mediterranean Sea waters surrounding the Balearic Archipelago, Spain [157]. *Cladosporium halotolerans* was also isolated from hypersaline water and detected on dolphin skin [158].

*Conidiobolus coronatus*, known to cause granulomatous infections, has been reported in dolphins and other mammals. These infections typically originate in the inferior turbinate and spread to involve facial and subcutaneous tissues and paranasal sinuses in humans [159].

*Sporothrix schenckii*, the causative agent of sporotrichosis, was identified in a Pacific white-sided dolphin, resulting in severe necrotizing granulomatous lymphadenitis. This diagnosis was made through histopathologic and electron microscopic studies, as well as fluorescent antibody techniques [160]. The presence of these fungi in cetaceans suggests a potential zoonotic risk for workers manipulating these animals, as these fungi could be transmitted by direct inoculation.

Finally, a concise overview of major fungal pathogens affecting cetaceans, as previously described, their characteristics, and implications for both cetacean health and potential zoonotic transmission can be consulted in Table 1.

## 6. Conclusions

To conclude, this review emphasizes the pressing need to enhance our knowledge and response to fungal infections in cetaceans, especially considering the limited information available and the potential zoonotic role of these shared pathogens in human populations. The critical insights gained from studying fungal pathogens in these marine mammals reveal a complex picture of ecological and health interdependencies, extending to human populations. The changing epidemiology and emerging patterns of fungal pathogenicity, coupled with the evolving resistance to antifungal treatments, call for a multifaceted One Health approach in research and veterinary and human healthcare practices.

Emphasizing cetaceans’ roles as hosts and potential vectors for these pathogens highlights the critical need for concerted efforts in ecological conservation and public health. This perspective is vital amidst the ongoing environmental changes influencing the transmission of infectious diseases. Future research should focus on the ecological determinants of fungal diseases, including the impact of human activities on the environment, to enhance our predictive capabilities regarding disease outbreaks. Moreover, developing cetacean-specific diagnostic and treatment strategies is imperative to address their unique physiological needs and the escalating challenge of antifungal resistance. In doing so, we will not only protect human health but also contribute significantly to the conservation of cetaceans, recognizing their indispensable role in maintaining marine ecosystem health and biodiversity.

## Figures and Tables

**Table 1:** Impact and zoonotic potential of some fungal pathogens in cetaceans.

Fungal Genus	Characteristics	Impact on Cetaceans	Zoonotic Potential
*Blastomyces*	Dimorphic fungi, historically endemic to certain regions but now expanding geographically	Cases reported in dolphins and other marine mammals; can cause systemic infections	Documented zoonotic transmission from marine mammals to humans
*Histoplasma*	Associated with soil enriched by avian or bat droppings; expanding due to climate and land use changes	Histoplasmosis in cetaceans similar to human cases, with potential for both pulmonary and disseminated forms	Potential zoonotic transmission, especially in coastal areas
*Coccidioides*	Endemic to arid soils, resilient in saline environments; expanding geographically	Documented infections in marine mammals, including severe cases in dolphins	Zoonotic implications due to environmental adaptability and expansion
*Paracoccidioides*	Causes paracoccidioidomycosis ceti; distinct species identified in cetaceans and humans	Nodular or verrucous lesions on dolphins; taxonomic distinctions indicate complex zoonotic implications	Limited direct transmission, but zoonotic aspects need careful consideration
*Candida*	Commensals that can become pathogenic; notable for antifungal resistance	Infections range from superficial to systemic; increasing reports in both captive and wild cetaceans	Possible zoonotic transmission, especially in captive environments
*Fusarium*	Opportunistic pathogens; notable in agriculture and human medicine	Cutaneous to systemic infections reported in cetaceans; challenging due to drug resistance	Environmental presence suggests zoonotic transmission risk
*Aspergillus*	Ubiquitous fungi causing a range of infections; issue of antifungal resistance	Various species causing infections in cetaceans, ranging from pulmonary to systemic	Zoonotic potential, especially given similar pathogenic patterns in humans and cetaceans
*Cryptococcus*	Causes cryptococcosis; thrives in diverse environments	Non-typical colonizers in cetaceans but linked to invasive diseases with high mortality	Zoonotic aspect significant, with potential for outbreaks affecting multiple species
*Malassezia*	Commonly found as commensals; associated with dermatological conditions	No direct evidence in cetaceans, but recent findings suggest potential presence and impact	Zoonotic importance due to shared environments and close contact
*Mucorales*	Opportunistic fungi causing severe infections in immunocompromised hosts	Emerging concern in cetaceans with reports of systemic and fatal infections	Indirect zoonotic risk due to environmental reservoirs; direct transmission less likely

## Data Availability

Not applicable.

## References

[1] Shanks S., van Schalkwyk M.C., Cunningham A.A. (2022). A call to prioritise prevention: Action is needed to reduce the risk of zoonotic disease emergence. Lancet Reg. Health Eur..

[2] Jones K.E., Patel N.G., Levy M.A., Storeygard A., Balk D., Gittleman J.L., Daszak P. (2008). Global trends in emerging infectious diseases. Nature.

[3] Plowright R.K., Parrish C.R., McCallum H., Hudson P.J., Ko A.I., Graham A.L., Lloyd-Smith J.O. (2017). Pathways to zoonotic spillover. Nat. Rev. Microbiol..

[4] Schmeller D.S., Courchamp F., Killeen G. (2020). Biodiversity loss, emerging pathogens and human health risks. Biodivers. Conserv..

[5] Lawler O.K., Allan H.L., Baxter P.W.J., Castagnino R., Tor M.C., Dann L.E., Hungerford J., Karmacharya D., Lloyd T.J., López-Jara M.J. (2021). The COVID-19 pandemic is intricately linked to biodiversity loss and ecosystem health. Lancet Planet. Health.

[6] Allen T., Murray K.A., Zambrana-Torrelio C., Morse S.S., Rondinini C., Di Marco M., Breit N., Olival K.J., Daszak P. (2017). Global hotspots and correlates of emerging zoonotic diseases. Nat. Commun..

[7] Rodrigues M.L., Nosanchuk J.D. (2020). Fungal diseases as neglected pathogens: A wake-up call to public health officials. PLoS Negl. Trop. Dis..

[8] Denning D.W. (2024). Global incidence and mortality of severe fungal disease. Lancet Infect. Dis..

[9] Blackwell M. (2011). The fungi: 1, 2, 3... 5.1 million species?. Am. J. Bot..

[10] Seyedmousavi S., Bosco S.M.G., de Hoog S., Ebel F., Elad D., Gomes R.R., Jacobsen I.D., Jensen H.E., Martel A., Mignon B. (2018). Fungal infections in animals: A patchwork of different situations. Med. Mycol..

[11] Garcia-Bustos V., Cabañero-Navalon M.D., Ruiz-Gaitán A., Salavert M., Tormo-Mas M.Á., Pemán J. (2023). Climate change, animals, and *Candida auris*: Insights into the ecological niche of a new species from a One Health approach. Clin. Microbiol. Infect..

[12] Waltzek T.B., Cortés-Hinojosa G., Wellehan J.F., Gray G.C. (2012). Marine mammal zoonoses: A review of disease manifestations. Zoonoses Public Health.

[13] Dolman S.J., Brakes P. (2018). Sustainable Fisheries Management and the Welfare of Bycaught and Entangled Cetaceans. Front. Vet. Sci..

[14] Ziarati M., Zorriehzahra M.J., Hassantabar F., Mehrabi Z., Dhawan M., Sharun K., Emran T.B., Dhama K., Chaicumpa W., Shamsi S. (2022). Zoonotic diseases of fish and their prevention and control. Vet. Q..

[15] Casadevall A. (2023). Global warming could drive the emergence of new fungal pathogens. Nat. Microbiol..

[16] He Q., Silliman B.R. (2019). Climate Change, Human Impacts, and Coastal Ecosystems in the Anthropocene. Curr. Biol..

[17] Větrovský T., Kohout P., Kopecký M., Machac A., Man M., Bahnmann B.D., Brabcová V., Choi J., Meszárošová L., Human Z.R. (2019). A meta-analysis of global fungal distribution reveals climate-driven patterns. Nat. Commun..

[18] Kumar V., Sarma V.V., Thambugala K.M., Huang J.J., Li X.Y., Hao G.F. (2021). Ecology and Evolution of Marine Fungi with Their Adaptation to Climate Change. Front. Microbiol..

[19] Garcia-Bustos V., Acosta-Hernández B., Cabañero-Navalón M.D., Pemán J., Ruiz-Gaitán A.C., Rosario Medina I. (2024). The Ecology of Non-Candida Yeasts and Dimorphic Fungi in Cetaceans: From Pathogenicity to Environmental and Global Health Implications. J. Fungi.

[20] Reif J.S., Peden-Adams M.M., Romano T.A., Rice C.D., Fair P.A., Bossart G.D. (2009). Immune dysfunction in Atlantic bottlenose dolphins (*Tursiops truncatus*) with lobomycosis. Med. Mycol..

[21] Van Bressem M.F., Raga J.A., Di Guardo G., Jepson P.D., Duignan P.J., Siebert U., Barrett T., Santos M.C., Moreno I.B., Siciliano S. (2009). Emerging infectious diseases in cetaceans worldwide and the possible role of environmental stressors. Dis. Aquat. Organ..

[22] Hess-Erga O.K., Moreno-Andrés J., Enger Ø., Vadstein O. (2019). Microorganisms in ballast water: Disinfection, community dynamics, and implications for management. Sci. Total Environ..

[23] Nøttestad L., Krafft B.A., Anthonypillai V., Bernasconi M., Langård L., Mørk H.L., Fernö A. (2015). Recent changes in distribution and relative abundance of cetaceans in the Norwegian Sea and their relationship with potential prey. Front. Ecol. Evolut..

[24] Schoch C.L., Ciufo S., Domrachev M., Hotton C.L., Kannan S., Khovanskaya R., Leipe D., Mcveigh R., O’Neill K., Robbertse B. (2020). NCBI Taxonomy: A comprehensive update on curation, resources and tools. Database.

[25] Sayers E.W., Cavanaugh M., Clark K., Ostell J., Pruitt K.D., Karsch-Mizrachi I. (2019). GenBank. Nucleic Acids Res..

[26] Mazi P.B., Rauseo A.M., Spec A. (2021). Blastomycosis. Infect. Dis. Clin. N. Am..

[27] Meece J.K., Anderson J.L., Fisher M.C., Henk D.A., Sloss B.L., Reed K.D. (2011). Population genetic structure of clinical and environmental isolates of Blastomyces dermatitidis, based on 27 polymorphic microsatellite markers. Appl. Environ. Microbiol..

[28] Brown E.M., McTaggart L.R., Zhang S.X., Low D.E., Stevens D.A., Richardson S.E. (2013). Phylogenetic analysis reveals a cryptic species *Blastomyces gilchristii*, sp. nov. within the human pathogenic fungus *Blastomyces dermatitidis*. PLoS ONE.

[29] Smith J.A., Riddell J., Kauffman C.A. (2013). Cutaneous manifestations of endemic mycoses. Curr. Infect. Dis. Rep..

[30] Cates M.B., Kaufman L., Grabau J.H., Pletcher J.M., Schroeder J.P. (1986). Blastomycosis in an Atlantic bottlenose dolphin. J. Am. Vet. Med. Assoc..

[31] Sweeney J.C., Migaki G., Vainik P.M., Conklin R.H. (1976). Systemic mycosis in marine mammals. J. Am. Vet. Med. Assoc..

[32] Zwick L.S., Briggs M.B., Tunev S.S., Lichtensteiger C.A., Murnane R.D. (2000). Disseminated blastomycosis in two California sea lions (*Zalophus californianus*). J. Zoo Wildl. Med..

[33] Migaki C., Jones S.R., Howard E.B. (1983). Mycotic diseases in marine mammals. Pathobiology of Marine Mammal Diseases.

[34] Williamson W.M., Lombard L.S., Getty R.E. (1959). North American blastomycosis in a northern sea lion. J. Am. Vet. Med. Assoc..

[35] Linz A.M., Anderson J.L., Meece J.K. (2024). Detection of *Blastomyces gilchristii* via metagenomic sequencing in outbreak-associated soils. Med. Mycol..

[36] Dukik K., Muñoz J.F., Jiang Y., Feng P., Sigler L., Stielow J.B., Freeke J., Jamalian A., Gerrits van den Ende B., McEwen J.G. (2017). Novel taxa of thermally dimorphic systemic pathogens in the *Ajellomycetaceae* (*Onygenales*). Mycoses.

[37] Azar M.M., Loyd J.L., Relich R.F., Wheat L.J., Hage C.A. (2020). Current Concepts in the Epidemiology, Diagnosis, and Management of Histoplasmosis Syndromes. Semin. Respir. Crit. Care Med..

[38] Araúz A.B., Papineni P. (2021). Histoplasmosis. Infect. Dis. Clin. N. Am..

[39] Ashraf N., Kubat R.C., Poplin V., Adenis A.A., Denning D.W., Wright L., McCotter O., Schwartz I.S., Jackson B.R., Chiller T. (2020). Re-drawing the Maps for Endemic Mycoses. Mycopathologia.

[40] Jensen E.D., Lipscomb T., Van Bonn B., Miller G., Fradkin J.M., Ridgway S.H. (1998). Disseminated histoplasmosis in an Atlantic bottlenose dolphin (*Tursiops truncatus*). J. Zoo Wildl. Med..

[41] Venn-Watson S., Daniels R., Smith C. (2012). Thirty year retrospective evaluation of pneumonia in a bottlenose dolphin *Tursiops truncatus* population. Dis. Aquat. Organ..

[42] Enoch D.A., Yang H., Aliyu S.H., Micallef C. (2017). The Changing Epidemiology of Invasive Fungal Infections. Methods Mol. Biol..

[43] Crum N.F. (2022). Coccidioidomycosis: A Contemporary Review. Infect Dis. Ther..

[44] Bays D.J., Thompson G.R. (2021). Coccidioidomycosis. Infect. Dis. Clin. N. Am..

[45] Shubitz L.F. (2007). Comparative aspects of coccidioidomycosis in animals and humans. Ann. N. Y. Acad. Sci..

[46] Thompson G.R., Pasqualotto A.C. (2021). Endemic mycoses: Expansion of traditional geographic ranges and pitfalls in management. Mycoses.

[47] Reidarson T.H., Harrell J.H., Rinaldi M.G., McBain J. (1998). Bronchoscopic and serologic diagnosis of *Aspergillus fumigatus* pulmonary infection in a bottlenose dolphin (*Tursiops truncatus*). J. Zoo Wildl. Med..

[48] Huckabone S.E., Gulland F.M., Johnson S.M., Colegrove K.M., Dodd E.M., Pappagianis D., Dunkin R.C., Casper D., Carlson E.L., Sykes J.E. (2015). Coccidioidomycosis and other systemic mycoses of marine mammals stranding along the central California, USA coast: 1998–2012. J. Wildl. Dis..

[49] Kanegae H., Sano A., Okubo-Murata M., Watanabe A., Tashiro R., Eto T., Ueda K., Hossain M.A., Itano E.N. (2022). Seroprevalences Against *Paracoccidioides cetii*: A Causative Agent for Paracoccidiomycosis Ceti (PCM-C) and *Coccidioides posadasii*; for Coccidioidomycosis (CCM) in Dall’s Porpoise (*Phocoenoides dalli*) and Harbor Porpoise (*Phocoena phocoena*) Stranded at Hokkaido, Japan. Mycopathologia.

[50] Vilela R., Huebner M., Vilela C., Vilela G., Pettersen B., Oliveira C., Mendoza L. (2021). The taxonomy of two uncultivated fungal mammalian pathogens is revealed through phylogeny and population genetic analyses. Sci. Rep..

[51] Paniz-Mondolfi A., Talhari C., Sander Hoffmann L., Connor D.L., Talhari S., Bermudez-Villapol L., Hernandez-Perez M., Van Bressem M.F. (2012). Lobomycosis: An emerging disease in humans and delphinidae. Mycoses..

[52] Vilela R., de Hoog S., Bensch K., Bagagli E., Mendoza L. (2023). A taxonomic review of the genus Paracoccidioides, with focus on the uncultivable species. PLoS Negl. Trop. Dis..

[53] Lobo J. (1931). Um caso de blastomicose specie o por uma specie nova, encontrada no Recife. Rev. Med..

[54] Migaki G., Valerio M.G., Irvine B., Garner F.M. (1971). Lobo’s disease in an Atlantic bottlenosed dolphin. J. Am. Vet. Med. Assoc..

[55] Symmers W.S. (1983). A possible case of Lôbo’s disease acquired in Europe from a bottle-nosed dolphin (*Tursiops truncatus*). Bull. Soc. Pathol. Exot. Filiales.

[56] Norton S.A. (2006). Dolphin-to-human transmission of lobomycosis?. J. Am. Acad. Dermatol..

[57] Reif J.S., Schaefer A.M., Bossart G.D. (2013). Lobomycosis: Risk of zoonotic transmission from dolphins to humans. Vector Borne Zoonotic Dis..

[58] Bossart G.D., Schaefer A.M., McCulloch S., Goldstein J., Fair P.A., Reif J.S. (2015). Mucocutaneous lesions in free-ranging Atlantic bottlenose dolphins *Tursiops truncatus* from the southeastern USA. Dis. Aquat. Organ..

[59] Daura-Jorge F.G., Simões-Lopes P.C. (2011). Lobomycosis-like disease in wild bottlenose dolphins *Tursiops truncatus* of Laguna, southern Brazil: Monitoring of a progressive case. Dis. Aquat. Organ..

[60] Bessesen B.L., Oviedo L., Burdett Hart L., Herra-Miranda D., Pacheco-Polanco J.D., Baker L., Saborío-Rodriguez G., Bermúdez-Villapol L., Acevedo-Gutiérrez A. (2014). Lacaziosis-like disease among bottlenose dolphins *Tursiops truncatus* photographed in Golfo Dulce, Costa Rica. Dis. Aquat. Organ..

[61] Bermúdez L., Van Bressem M.F., Reyes-Jaimes O., Sayegh A.J., Paniz-Mondolfi A.E. (2009). Lobomycosis in man and lobomycosis-like disease in bottlenose dolphin, Venezuela. Emerg. Infect. Dis..

[62] de Moura J.F., Hauser-Davis R.A., Lemos L., Emin-Lima R., Siciliano S. (2014). Guiana dolphins (*Sotalia guianensis*) as marine ecosystem sentinels: Ecotoxicology and emerging diseases. Rev. Environ. Contam. Toxicol..

[63] Esperón F., García-Párraga D., Bellière E.N., Sánchez-Vizcaíno J.M. (2012). Molecular diagnosis of lobomycosis-like disease in a bottlenose dolphin in captivity. Med. Mycol..

[64] Kiszka J., Van Bressem M.F., Pusineri C. (2009). Lobomycosis-like disease and other skin conditions in Indo-Pacific bottlenose dolphins *Tursiops aduncus* from the Indian Ocean. Dis. Aquat. Organ..

[65] Lane E.P., de Wet M., Thompson P., Siebert U., Wohlsein P., Plön S. (2014). A systematic health assessment of Indian Ocean bottlenose (*Tursiops aduncus*) and Indo-Pacific humpback (*Sousa plumbea*) dolphins incidentally caught in shark nets off the KwaZulu-Natal Coast, South Africa. PLoS ONE.

[66] Minakawa T., Ueda K., Tanaka M., Tanaka N., Kuwamura M., Izawa T., Konno T., Yamate J., Itano E.N., Sano A. (2016). Detection of Multiple Budding Yeast Cells and a Partial Sequence of 43-kDa Glycoprotein Coding Gene of Paracoccidioides brasiliensis from a Case of Lacaziosis in a Female Pacific White-Sided Dolphin (*Lagenorhynchus obliquidens*). Mycopathologia.

[67] Grotta G., Couppie P., Demar M., Alsibai K.D., Blaizot R. (2023). Fungal Density in Lobomycosis in French Guiana: A Proposal for a New Clinico-Histological and Therapeutic Classification. *J. Fungi*
**2023**, *9*, 1005. J. Fungi.

[68] Ueda K., Sano A., Yamate J., Itano E.N., Kuwamura M., Izawa T., Tanaka M., Hasegawa Y., Chibana H., Izumisawa Y. (2013). Two cases of lacaziosis in bottlenose dolphins (*Tursiops truncatus*) in Japan. Case Rep. Vet. Med..

[69] Reif J.S., Mazzoil M.S., McCulloch S.D., Varela R.A., Goldstein J.D., Fair P.A., Bossart G.D. (2006). Lobomycosis in Atlantic bottlenose dolphins from the Indian River Lagoon, Florida. J. Am. Vet. Med. Assoc..

[70] Rotstein D.S., West K., Levine G., Lockhart S.R., Raverty S., Morshed M.G., Rowles T. (2010). *Cryptococcus gattiivgi* in a spinner dolphin (*Stenella longirostris*) from Hawaii. J. Zoo Wildl. Med..

[71] Grotta G., Couppie P., Demar M., Drak Alsibai K., Blaizot R. (2023). Fungal Density in Lobomycosis in French Guiana: A Proposal for a New Clinico-Histological and Therapeutic Classification. J. Fungi.

[72] Thomas-Rüddel D.O., Schlattmann P., Pletz M., Kurzai O., Bloos F. (2022). Risk Factors for Invasive Candida Infection in Critically Ill Patients: A Systematic Review and Meta-analysis. Chest.

[73] d’Enfert C., Kaune A.K., Alaban L.R., Chakraborty S., Cole N., Delavy M., Kosmala D., Marsaux B., Fróis-Martins R., Morelli M. (2021). The impact of the Fungus-Host-Microbiota interplay upon Candida albicans infections: Current knowledge and new perspectives. FEMS Microbiol. Rev..

[74] Pristov K.E., Ghannoum M.A. (2019). Resistance of *Candida* to azoles and echinocandins worldwide. Clin. Microbiol. Infect..

[75] McCarty T.P., White C.M., Pappas P.G. (2021). Candidemia and Invasive Candidiasis. Infect. Dis. Clin. N. Am..

[76] Barantsevich N., Barantsevich E. (2022). Diagnosis and Treatment of Invasive Candidiasis. Antibiotics.

[77] Pappas P.G., Lionakis M.S., Arendrup M.C., Ostrosky-Zeichner L., Kullberg B.J. (2018). Invasive candidiasis. Nat. Rev. Dis. Prim..

[78] Garcia-Bustos V., Cabanero-Navalon M.D., Ruiz-Saurí A., Ruiz-Gaitán A.C., Salavert M., Tormo M.Á., Pemán J. (2021). What Do We Know about *Candida auris*? State of the Art, Knowledge Gaps, and Future Directions. Microorganisms.

[79] Paiva J.A., Pereira J.M. (2023). Treatment of invasive candidiasis in the era of *Candida* resistance. Curr. Opin. Crit. Care.

[80] Gómez-López A. (2020). Antifungal therapeutic drug monitoring: Focus on drugs without a clear recommendation. Clin. Microbiol. Infect..

[81] Ohno Y., Akune Y., Inoshima Y., Kano R. (2019). First isolation of voriconazole-resistant *Candida albicans*, *C. tropicalis*, and *Aspergillus niger* from the blowholes of bottlenose dolphins (*Tursiops truncatus*). J. Vet. Med. Sci..

[82] Nakeeb S., Targowski S.P., Spotte S. (1977). Chronic cutaneous candidiasis in bottle-nosed dolphins. J. Am. Vet. Med. Assoc..

[83] Dunn J.L., Buck J.D., Spotte S. (1982). Candidiasis in captive cetaceans. J. Am. Vet. Med. Assoc..

[84] Fothergill M., Jogessar V.B. (1986). Hematological changes in two *Lagenorhynchus obscurus* treated with. Aquat. Mamm..

[85] Takahashi H., Ueda K., Itano E.N., Yanagisawa M., Murata Y., Murata M., Yaguchi T., Murakami M., Kamei K., Inomata T. (2010). *Candida albicans* and *C. tropicalis* Isolates from the Expired Breathes of Captive Dolphins and Their Environments in an Aquarium. Vet. Med. Int..

[86] Lee C., Jensen E.D., Meegan J., Ivančić M., Bailey J., Hendrickson D., Weiss J., Grindley J., Costidis A.M., Wisbach G. (2019). Surgical Management of a Chronic Neck Abscess in a U.S. Navy Bottlenose Dolphin. Mil. Med..

[87] Nollens H.H., Wellehan J.F., Saliki J.T., Caseltine S.L., Jensen E.D., Van Bonn W., Venn-Watson S. (2008). Characterization of a parainfluenza virus isolated from a bottlenose dolphin (*Tursiops truncatus*). Vet. Microbiol..

[88] Buck J.D., Overstrom N.A., Patton G.W., Anderson H.F., Gorzelany J.F. (1991). Bacteria associated with stranded cetaceans from the northeast USA and southwest Florida Gulf coasts. Dis. Aquat. Org..

[89] Haulena M., Huff D., Ivančić M., Muhammad M., Hoang L., Zabek E., Raverty S. (2010). Intestinal torsion secondary to chronic candidiasis caused by *Candida krusei* in a Pacific white-sided dolphin (*Lagenorhynchus obliquidens*). Proc. Int. Assoc. Aquat. Anim. Med..

[90] Schmid J., Hunter P.R., White G.C., Nand A.K., Cannon R.D. (1995). Physiological traits associated with success of Candida albicans strains as commensal colonizers and pathogens. J. Clin. Microbiol..

[91] Shotts E.B., Albert T.F., Wooley R.E., Brown J. (1990). Microflora associated with the skin of the bowhead whale (*Balaena mysticetus*). J. Wildl. Dis..

[92] Buck J.D., Bubucis P.M., Spotte S. (1988). Microbiological characterization of three Atlantic whiteside dolphins (*Lagenorhynchus acutus*) from stranding through captivity with subsequent rehabilitation and release of one animal. Zoo Biol..

[93] Morris P.J., Johnson W.R., Pisani J., Bossart G.D., Adams J., Reif J.S., Fair P.A. (2011). Isolation of culturable microorganisms from free-ranging bottlenose dolphins (*Tursiops truncatus*) from the southeastern United States. Vet. Microbiol..

[94] Buck J.D. (1980). Occurrence of human-associated yeasts in the feces and pool waters of captive bottlenosed dolphins (*Tursiops truncatus*). J. Wildl. Dis..

[95] Hof H. (2020). The Medical Relevance of *Fusarium* spp. J. Fungi.

[96] Nucci M., Anaissie E. (2023). Invasive fusariosis. Clin. Microbiol. Rev..

[97] Frasca S., Dunn J.L., Cooke J.C., Buck J.D. (1996). Mycotic dermatitis in an Atlantic white-sided dolphin, a pygmy sperm whale, and two harbor seals. J. Am. Vet. Med. Assoc..

[98] Naples L.M., Poll C.P., Berzins I.K. (2012). Successful treatment of a severe case of fusariomycosis in a beluga whale (*Delphinapterus leucas leucas*). J. Zoo Wildl. Med..

[99] Tanaka M., Izawa T., Kuwamura M., Nakao T., Maezono Y., Ito S., Murata M., Murakami M., Sano A., Yamate J. (2012). Deep granulomatous dermatitis of the fin caused by *Fusarium solani* in a false killer whale (*Pseudorca crassidens*). J. Vet. Med. Sci..

[100] Staggs L., St Leger J., Bossart G., Townsend F.I., Hicks C., Rinaldi M. (2010). A novel case of *Fusarium oxysporum* infection in an Atlantic bottlenose dolphin (*Tursiops truncatus*). J. Zoo Wildl. Med..

[101] Kohata E., Kano R., Akune Y., Ohno Y., Soichi M., Yanai T., Hasegawa A., Kamata H. (2013). Environmental isolates of fungi from aquarium pools housing killer whales (*Orcinus orca*). Mycopathologia.

[102] Thompson G.R., Young J.H. (2021). *Aspergillus* Infections. N. Engl. J. Med..

[103] Latgé J.P., Chamilos G. (2019). *Aspergillus fumigatus* and Aspergillosis in 2019. Clin. Microbiol. Rev..

[104] Kanaujia R., Singh S., Rudramurthy S.M. (2023). Aspergillosis: An Update on Clinical Spectrum, Diagnostic Schemes, and Management. Curr. Fungal Infect. Rep..

[105] Pérez-Cantero A., López-Fernández L., Guarro J., Capilla J. (2020). Azole resistance mechanisms in *Aspergillus*: Update and recent advances. Int. J. Antimicrob. Agents.

[106] Elad D., Segal E. (2018). Diagnostic Aspects of Veterinary and Human Aspergillosis. Front. Microbiol..

[107] Groch K.R., Díaz-Delgado J., Sacristán C., Oliveira D.E., Souza G., Sánchez-Sarmiento A.M., Costa-Silva S., Marigo J., Castilho P.V., Cremer M.J. (2018). Pulmonary and systemic fungal infections in an Atlantic spotted dolphin and a Bryde’s whale, Brazil. Dis. Aquat. Organ..

[108] Balik S.E., Ossiboff R.J., Stacy N.I., Wellehan J.F.X., Huguet E.E., Gallastegui A., Childress A.L., Baldrica B.E., Dolan B.A., Adler L.E. (2023). Case report: *Sarcocystis speeri*, *Aspergillus fumigatus*, and novel *Treponema* sp. infections in an adult Atlantic spotted dolphin (*Stenella frontalis*). Front. Vet. Sci..

[109] Hamel P.E.S., Giglio R.F., Cassle S.E., Farina L.L., Leone A.M., Walsh M.T. (2020). Postmortem computed tomography and magnetic resonance imaging findings in a case of coinfection of dolphin morbillivirus and *Aspergillus fumigatus* in a juvenile bottlenose dolphin (*Tursiops truncatus*). J. Zoo Wildl. Med..

[110] Cassle S.E., Landrau-Giovannetti N., Farina L.L., Leone A., Wellehan J.F., Stacy N.I., Thompson P., Herring H., Mase-Guthrie B., Blas-Machado U. (2016). Coinfection by *Cetacean morbillivirus* and *Aspergillus fumigatus* in a juvenile bottlenose dolphin (*Tursiops truncatus*) in the Gulf of Mexico. J. Vet. Diagn. Investig..

[111] Ohno Y., Akune Y., Nitto H., Inoshima Y. (2019). Leukopenia induced by micafungin in a bottlenose dolphin (*Tursiops truncatus*): A case report. J. Vet. Med. Sci..

[112] Delaney M.A., Terio K.A., Colegrove K.M., Briggs M.B., Kinsel M.J. (2013). Occlusive fungal tracheitis in 4 captive bottlenose dolphins (*Tursiops truncatus*). Vet. Pathol..

[113] Desoubeaux G., Le-Bert C., Fravel V., Clauss T., Delaune A.J., Soto J., Jensen E.D., Flower J.E., Wells R., Bossart G.D. (2018). Evaluation of a genus-specific ELISA and a commercial Aspergillus Western blot IgG^®^ immunoblot kit for the diagnosis of aspergillosis in common bottlenose dolphins (*Tursiops truncatus*). Med. Mycol..

[114] Bunskoek P.E., Seyedmousavi S., Gans S.J., van Vierzen P.B., Melchers W.J., van Elk C.E., Mouton J.W., Verweij P.E. (2017). Successful treatment of azole-resistant invasive aspergillosis in a bottlenose dolphin with high-dose posaconazole. Med. Mycol. Case Rep..

[115] Abdo W., Kawachi T., Sakai H., Fukushi H., Kano R., Shibahara T., Shirouzu H., Kakizoe Y., Tuji H., Yanai T. (2012). Disseminated mycosis in a killer whale (*Orcinus orca*). J. Vet. Diagn. Investig..

[116] Prahl S., Jepson P.D., Sanchez-Hanke M., Deaville R., Siebert U. (2011). Aspergillosis in the middle ear of a harbour porpoise (*Phocoena phocoena*): A case report. Mycoses.

[117] Seibel H., Beineke A., Siebert U. (2010). Mycotic otitis media in a harbour porpoise (*Phocoena phocoena*). J. Comp. Pathol..

[118] Dagleish M.P., Patterson I.A., Foster G., Reid R.J., Linton C., Buxton D. (2006). Intracranial granuloma caused by asporogenic *Aspergillus fumigatus* in a harbour porpoise (*Phocoena phocoena*). Vet. Rec..

[119] Barley J., Foster G., Reid B., Dagleish M., Howie F. (2007). Encephalitis in a northern bottlenose whale. Vet. Rec..

[120] Dagleish M.P., Foster G., Howie F.E., Reid R.J., Barley J. (2008). Fatal mycotic encephalitis caused by *Aspergillus fumigatus* in a northern bottlenose whale (*Hyperoodon ampullatus*). Vet. Rec..

[121] Domingo M., Visa J., Pumarola M., Marco A.J., Ferrer L., Rabanal R., Kennedy S. (1992). Pathologic and immunocytochemical studies of morbillivirus infection in striped dolphins (*Stenella coeruleoalba*). Vet. Pathol..

[122] Grattarola C., Giorda F., Iulini B., Pautasso A., Ballardini M., Zoppi S., Marsili L., Peletto S., Masoero L., Varello K. (2018). Occlusive mycotic tracheobronchitis and systemic *Alphaherpesvirus* coinfection in a free-living striped dolphin *Stenella coeruleoalba* in Italy. Dis. Aquat. Organ..

[123] Hong S.B., Go S.J., Shin H.D., Frisvad J.C., Samson R.A. (2005). Polyphasic taxonomy of *Aspergillus fumigatus* and related species. Mycologia.

[124] Hong S.B., Kim D.H., Park I.C., Choi Y.J., Shin H.D., Samson R. (2010). Re-identification of *Aspergillus fumigatus* sensu lato based on a new concept of species delimitation. J. Microbiol..

[125] Gushiken A.C., Saharia K.K., Baddley J.W. (2021). Cryptococcosis. Infect. Dis. Clin. N. Am..

[126] Gullo F.P., Rossi S.A., Sardi Jde C., Teodoro V.L., Mendes-Giannini M.J., Fusco-Almeida A.M. (2013). Cryptococcosis: Epidemiology, fungal resistance, and new alternatives for treatment. Eur. J. Clin. Microbiol. Infect. Dis..

[127] Howard-Jones A.R., Sparks R., Pham D., Halliday C., Beardsley J., Chen S.C. (2022). Pulmonary Cryptococcosis. J. Fungi.

[128] Chang C.C., Chen S.C. (2015). Colliding Epidemics and the Rise of Cryptococcosis. J. Fungi.

[129] Danesi P., Falcaro C., Schmertmann L.J., de Miranda L.H.M., Krockenberger M., Malik R. (2021). Cryptococcus in Wildlife and Free-Living Mammals. J. Fungi.

[130] Chen S.C., Meyer W., Sorrell T.C. (2014). *Cryptococcus gattii* infections. Clin. Microbiol. Rev..

[131] Rathore S.S., Sathiyamoorthy J., Lalitha C., Ramakrishnan J. (2022). A holistic review on *Cryptococcus neoformans*. Microb. Pathog..

[132] Teman S.J., Gaydos J.K., Norman S.A., Huggins J.L., Lambourn D.M., Calambokidis J., Ford J.K.B., Hanson M.B., Haulena M., Zabek E. (2021). Epizootiology of a *Cryptococcus gattii* outbreak in porpoises and dolphins from the Salish Sea. Dis. Aquat. Organ..

[133] Stephen C., Lester S., Black W., Fyfe M., Raverty S. (2002). Multispecies outbreak of cryptococcosis on southern Vancouver Island, British Columbia. Can. Vet. J..

[134] Kidd S.E., Bach P.J., Hingston A.O., Mak S., Chow Y., MacDougall L., Kronstad J.W., Bartlett K.H. (2007). *Cryptococcus gattii* dispersal mechanisms, British Columbia, Canada. Emerg. Infect. Dis..

[135] Miller W.G., Padhye A.A., van Bonn W., Jensen E., Brandt M.E., Ridgway S.H. (2002). Cryptococcosis in a bottlenose dolphin (*Tursiops truncatus*) caused by *Cryptococcus neoformans* var. gattii. J. Clin. Microbiol..

[136] Gales N., Wallace G., Dickson J. (1985). Pulmonary cryptococcosis in a striped dolphin (*Stenella coeruleoalba*). J. Wildl. Dis..

[137] Norman S.A., Raverty S., Zabek E., Etheridge S., Ford J.K., Hoang L.M., Morshed M. (2011). Maternal-fetal transmission of *Cryptococcus gattii* in harbor porpoise. Emerg. Infect. Dis..

[138] Mouton M., Reeb D., Botha A., Best P. (2009). Yeast infection in a beached southern right whale (*Eubalaena australis*) neonate. J. Wildl. Dis..

[139] Fenton H., Daoust P.Y., Forzán M.J., Vanderstichel R.V., Ford J.K., Spaven L., Lair S., Raverty S. (2017). Causes of mortality of harbor porpoises *Phocoena phocoena* along the Atlantic and Pacific coasts of Canada. Dis. Aquat. Organ..

[140] Wan X.L., McLaughlin R.W., Zheng J.S., Hao Y.J., Fan F., Tian R.M., Wang D. (2018). Microbial communities in different regions of the gastrointestinal tract in East Asian finless porpoises (*Neophocaena asiaeorientalis sunameri*). Sci. Rep..

[141] Hobi S., Cafarchia C., Romano V., Barrs V.R. (2022). Malassezia: Zoonotic Implications, Parallels and Differences in Colonization and Disease in Humans and Animals. J. Fungi.

[142] Theelen B., Cafarchia C., Gaitanis G., Bassukas I.D., Boekhout T., Dawson T.L. (2018). Malassezia ecology, pathophysiology, and treatment. Med. Mycol..

[143] Velegraki A., Cafarchia C., Gaitanis G., Iatta R., Boekhout T. (2015). Malassezia infections in humans and animals: Pathophysiology, detection, and treatment. PLoS Pathog..

[144] Huang C.Y., Peng C.C., Hsu C.H., Chang J.H., Chiu N.C., Chi H. (2020). Systemic Infection Caused by *Malassezia pachydermatis* in Infants: Case Series and Review of the Literature. Pediatr. Infect. Dis. J..

[145] Guillot J., Petit T., Degorce-Rubiales F., Guého E., Chermette R. (1998). Dermatitis caused by *Malassezia pachydermatis* in a California sea lion (*Zalophus californianus*). Vet. Rec..

[146] Nakagaki K., Hata K., Iwata E., Takeo K. (2000). Malassezia pachydermatis isolated from a South American sea lion (*Otaria byronia*) with dermatitis. J. Vet. Med. Sci..

[147] Gupta I., Baranwal P., Singh G., Gupta V. (2023). Mucormycosis, past and present: A comprehensive review. Future Microbiol..

[148] Cornely O.A., Alastruey-Izquierdo A., Arenz D., Chen S.C.A., Dannaoui E., Hochhegger B., Hoenigl M., Jensen H.E., Lagrou K., Lewis R.E. (2019). Global guideline for the diagnosis and management of mucormycosis: An initiative of the European Confederation of Medical Mycology in cooperation with the Mycoses Study Group Education and Research Consortium. Lancet Infect. Dis..

[149] Alqarihi A., Kontoyiannis D.P., Ibrahim A.S. (2023). Mucormycosis in 2023: An update on pathogenesis and management. Front. Cell. Infect. Microbiol..

[150] Robeck T.R., Dalton L.M. (2002). *Saksenaea vasiformis* and *Apophysomyces elegans* zygomycotic infections in bottlenose dolphins (*Tursiops truncatus*), a killer whale (*Orcinus orca*), and pacific white-sided dolphins (*Lagenorhynchus obliquidens*). J. Zoo Wildl. Med..

[151] Bragulat M.R., Castellá G., Isidoro-Ayza M., Domingo M., Cabañes F.J. (2017). Characterization and phylogenetic analysis of a *Cunninghamella bertholletiae* isolate from a bottlenose dolphin (*Tursiops truncatus*). Rev. Iberoam. Micol..

[152] Isidoro-Ayza M., Pérez L., Cabañes F.J., Castellà G., Andrés M., Vidal E., Domingo M. (2014). Central nervous system mucormycosis caused by *Cunninghamella bertholletiae* in a bottlenose dolphin (*Tursiops truncatus*). J. Wildl. Dis..

[153] Chang L., Qi Y., Wang Y., Liu C.H., Chen S., Miao B., Tong D. (2021). Systemic mucormycosis caused by *Rhizopus microsporus* in a captive bottlenose dolphin. Vet. Med. Sci..

[154] Cerezo A., Quesada-Canales O., Sierra E., Díaz-Delgado J., Fernández A., Henningson J., Arbelo M. (2018). Pyogranulomatous obliterative laryngotracheitis by *Rhizopus arrhizus* (syn. *R. oryzae*) in a free-ranging Atlantic spotted dolphin *Stenella frontalis*. Dis Aquat Organ..

[155] Wünschmann A., Siebert U., Weiss R. (1999). Rhizopusmycosis in a harbor porpoise from the Baltic Sea. J. Wildl. Dis..

[156] Marques G.N., Silva N.U., Leal M.O., Flanagan C.A. (2021). The use of posaconazole delayed-release tablets in the successful treatment of suspected mucormycosis in a bottlenose dolphin (*Tursiops truncatus*) calf. Med Mycol Case Rep..

[157] Hermosilla C., Hirzmann J., Silva L.M.R., Brotons J.M., Cerdà M., Prenger-Berninghoff E., Ewers C., Taubert A. (2018). Occurrence of anthropozoonotic parasitic infections and faecal microbes in free-ranging sperm whales (*Physeter macrocephalus*) from the Mediterranean Sea. Parasitol. Res..

[158] Zalar P., de Hoog G.S., Schroers H.J., Crous P.W., Groenewald J.Z., Gunde-Cimerman N. (2007). Phylogeny and ecology of the ubiquitous saprobe *Cladosporium sphaerospermum*, with descriptions of seven new species from hypersaline environments. Stud. Mycol..

[159] Gugnani H.C. (1992). Entomophthoromycosis due to *Conidiobolus*. Eur. J. Epidemiol..

[160] Migaki G., Font R.L., Kaplan W., Asper E.D. (1978). Sporotrichosis in a Pacific white-sided dolphin (*Lagenorhynchus obliquidens*). Am. J. Vet. Res..

